# Radiation Recall Pneumonitis: The Open Challenge in Differential Diagnosis of Pneumonia Induced by Oncological Treatments

**DOI:** 10.3390/jcm12041442

**Published:** 2023-02-10

**Authors:** Francesca Grassi, Vincenza Granata, Roberta Fusco, Federica De Muzio, Carmen Cutolo, Michela Gabelloni, Alessandra Borgheresi, Ginevra Danti, Carmine Picone, Andrea Giovagnoni, Vittorio Miele, Nicoletta Gandolfo, Antonio Barile, Valerio Nardone, Roberta Grassi

**Affiliations:** 1 Division of Radiology, Università Degli Studi Della Campania Luigi Vanvitelli, 80127 Naples, Italy; 2 Italian Society of Medical and Interventional Radiology (SIRM), SIRM Foundation, Via della Signora 2, 20122 Milan, Italy; 3 Division of Radiology, Istituto Nazionale Tumori IRCCS Fondazione Pascale—IRCCS di Napoli, 80131 Naples, Italy; 4 Medical Oncology Division, Igea SpA, 80015 Naples, Italy; 5 Diagnostic Imaging Section, Department of Medical and Surgical Sciences & Neurosciences, University of Molise, 86100 Campobasso, Italy; 6 Department of Medicine, Surgery and Dentistry, University of Salerno, 84084 Salerno, Italy; 7 Department of Translational Research, Diagnostic and Interventional Radiology, University of Pisa, 56126 Pisa, Italy; 8 Department of Clinical, Special and Dental Sciences, University Politecnica Delle Marche, Via Conca 71, 60126 Ancona, Italy; 9 Department of Radiology, University Hospital “Azienda Ospedaliera Universitaria delle Marche”, Via Conca 71, 60126 Ancona, Italy; 10 Department of Radiology, Careggi University Hospital, Largo Brambilla 3, 50134 Florence, Italy; 11 Diagnostic Imaging Department, Villa Scassi Hospital-ASL 3, Corso Scassi 1, 16149 Genoa, Italy; 12 Department of Applied Clinical Sciences and Biotechnology, University of L’Aquila, Via Vetoio 1, 67100 L’Aquila, Italy

**Keywords:** radiation treatment, imaging, computed tomography, radiation recall pneumonitis

## Abstract

The treatment of primary and secondary lung neoplasms now sees the fundamental role of radiotherapy, associated with surgery and systemic therapies. The improvement in survival outcomes has also increased attention to the quality of life, treatment compliance and the management of side effects. The role of imaging is not only limited to recognizing the efficacy of treatment but also to identifying, as soon as possible, the uncommon effects, especially when more treatments, such as chemotherapy, immunotherapy and radiotherapy, are associated. Radiation recall pneumonitis is an uncommon treatment complication that should be correctly characterized, and it is essential to recognize the mechanisms of radiation recall pneumonitis pathogenesis and diagnostic features in order to promptly identify them and adopt the best therapeutic strategy, with the shortest possible withdrawal of the current oncological drug. In this setting, artificial intelligence could have a critical role, although a larger patient data set is required.

## 1. Introduction

Lung cancer is one of the most common tumors, ranking second place in terms of incidence rate and first in mortality rate [1]. In addition, the lungs remain a metastatic disease common site for primary lung cancer and patients with extrathoracic tumors [2]. Although the possibility that a patient is fit for surgical resection is correlated to the disease stage, radiotherapy (RT) is a standard of care for advanced or non-operable lung cancer patients or oligometastatic patients [3,4,5,6,7,8,9,10]. Nowadays, the treatment of lung tumors is complex, involving multiple therapies, such as surgery, systemic therapies (chemotherapy-targeted agents and immunotherapy) or ablation treatment [11,12,13,14,15,16,17,18,19,20]; however, RT is the only modality for which there are indications in all stages of disease and across all categories of patient performance status as an only modality or in association with others [21,22,23,24].

In recent years, it has been possible to guarantee a highly conformal radiotherapy treatment plan thanks to use of intensity-modulated radiotherapy (IMRT) and volumetric- modulated arc therapy (VMAT), image guidance radiotherapy (IGRT) and modern respiratory–motion management [25]. In addition, the increased survival of cancer patients has led to increased attention on adjacent organs at risk (OARs). This leads to higher savings of the organs surrounding the disease.

Despite the clear RT advantages both in terms of overall survival (OS) and the patient’s quality of life, the possibility of the use of a trimodal approach for primary or metastatic malignancies has notably increased [26,27,28,29]; however, the combination of more strategies may also lead to an increase in side effects, which should be identified as soon as possible.

One of the main side effects that could occur is pneumonitis, which should be characterized as related to the treatment or a possible infection in an immunocompromised patient. This complication, also, should be differentiated from a new lesion to avoid a misdiagnosis of progression disease.

Regarding pneumonia related to cancer therapy, it is possible to identify three main causes: it is induced by drugs, radiotherapy and the combination of both.

Drug-induced pneumonia may be related to cytotoxic drugs, such as gemcitabine [30], target therapies (e.g., everolimus) [31,32] or checkpoint inhibitors (ICIs) that modulate the immunological response (e.g., nivolumab, atezolizumab, pembrolizumab, ipilimumab), which can cause checkpoint inhibitors pneumonitis (CIP) [33].

Radiation pneumonitis (RP) and radiation fibrosis (RF) are, respectively, two examples of acute and chronic damage. RP and RF occur in 5–20% of patients treated with radiotherapy and are generally dose-limiting toxicities [34]. The diagnosis is based on the combination of several elements, such as the evaluation of radiotherapy treatment planning, diagnostic imaging pre and post radiotherapy and the exclusion of other lung diseases, such as infections. Treatment is recommended only when symptoms appear [35].

Radiation recall is an acute inflammatory reaction following the administration of a drug confined to a previous radio-treated area, which can arise several weeks or months after the end of radiotherapy, involving different anatomical districts, including skin, the gastroenteric tract and lungs [36]. Usually, the reaction is resolved with drug suspension and the use of steroids. Radiation recall pneumonitis (RRP) is, therefore, a lung inflammatory reaction, determined by the interaction of at least two agents, and it is necessary to differentiate RRP from pneumonia induced by individual agents or a new lesion for proper patient management.

In this narrative review, we report the damage underlying mechanisms, the diagnostic management and the differential diagnoses for patient risk stratification and proper management.

## 2. Mechanisms of Lung Radiation Damage

**Figure 1:**
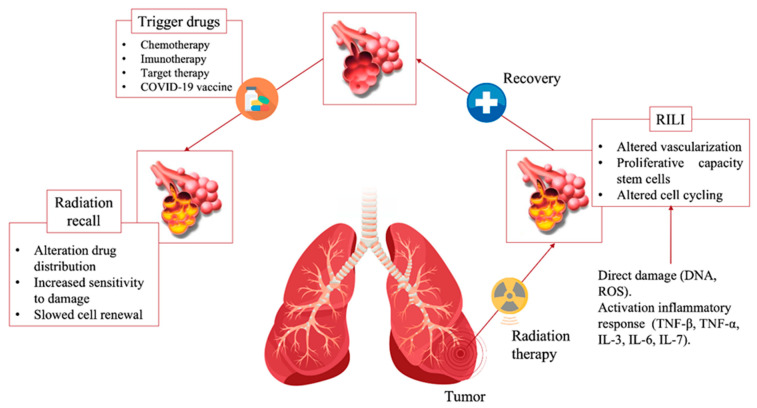
Schematic representation of radiation recall pneumonitis. RILI = radiation-induced lung injury.

The underlying mechanisms of lung radiation damage are well known and lead to acute and chronic damage [37]. Radiation causes direct damage to the DNA and indirect damage through the production of reactive oxygen species (ROS), causing changes in vascularity and capillary permeability, activation of the inflammatory response and alteration of immunological response (Figure 1) [38]. In most cases, radiation-induced damage tends to resolve over the course of a few weeks, either spontaneously or with corticosteroids therapy. Conversely, continuous production of cytokines and the procrastination of inflammatory responses determine excessive fibroblast proliferation resulting on fibrosis areas [39].

Radiation recall, on the other hand, is the reactivation of the damage at the level of the previously treated site, at a distance of several times from the radiotherapy treatment, following the administration of a drug. The phenomenon can occur following the administration of chemotherapy [40,41], target therapy [42,43], immunotherapies [44,45] or vaccinations [46,47], and it is not radiation dose-related. Radiation recall reactions were first described in 1959 by D’Angio et al. who described the onset of dermatitis in patients treated with actinomycin D, after having previously undergone radiotherapy treatments, demonstrating the absence of such reactions after only treatment with actinomycin and the significant increase of the same in patients treated with the combination of actinomycin and radiotherapy compared to those treated exclusively with radiotherapy [48]. Radiation recall can be an unpredictable subclinical or clinical event, even severe, that can impact the continuity of patients’ systemic care. Radiation recall is enigmatic with histopathological and clinical features of acute or chronic inflammation, self-limiting once the trigger drug is stopped. Rechallenge with drug does not necessarily cause reactivation of the reaction.

The underlying mechanisms are not yet well known but can be researched in the pathophysiological alterations induced by radiotherapy and subsequently by the drug administered. Various hypotheses have been proposed as to the mechanism of RRR; a non-immune fixed drug reaction-like condition, a dysregulated release of reactive oxygen species, abnormalities of tissue vasculature and impaired DNA repair. Alteration of the proliferative capacity of stem cells in the irradiated tissue causes increased sensitivity to the cytotoxic drug. In addition, the depletion of progenitor cells and increased cell cycle lead to increased susceptibility to drugs that inhibit cell renewal. The previous radiation-induced inflammatory response may lead to an impaired immune response which may lead to a hypersensitivity reaction at the level of the previously irradiated area, following the administration of immunostimulant drugs, such as immunotherapy [49] or vaccines [50].

## 3. Diagnostic Management

During RT treatments, imaging is employed in different phases: planning, supervising and treatment response assessment, including complications [51,52,53,54,55,56,57,58,59,60,61,62,63,64,65,66,67,68,69,70].

With regard to “technique efficacy”, it refers to the “complete necrosis” of the tumor; a feature that may be confirmed with imaging follow-up at precise time points [61]. Complications refer to any unexpected variation from a procedural course or adverse events that include any possible damage correlated to the treatment; these events should be analyzed according to the severity and occurrence time (early or late) [60,61]. In post treatment phases, different imaging tolls may be employed, alone or in combination [71,72,73,74,75,76].

With regard to treatment-related pneumonitis chest radiography, due to its low specificity, it can be used as a screening investigation, while the role of PET is still unclear due to the potential overlap of the metabolic activity of infectious processes with malignant tumors. Chest CT allows early parenchymal changes and their evolution to be evaluated, thus representing the gold standard for the study of lung parenchyma [77,78,79,80,81,82,83,84,85,86,87,88]. Nevertheless, diagnosis is complicated due to the overlapping of the various CT patterns, and in this scenario, we should consider radiation pneumonia (RP), pneumonia by immune checkpoint inhibitors (CIP) and the possibility that the lung involvement is due to disease progression that may be manifested as pulmonary lymphangitis [89]. In addition to these, a differential diagnosis must also be undertaken regarding infectious pneumonia. Regarding this last point, it is necessary to also focus on COVID-19 pneumonia [90,91,92,93,94,95,96,97,98,99,100,101,102,103,104,105,106] and RRP trigged by COVID-19 vaccinations [107,108], considering the pandemic conditions.

### 3.1. Radiological and Clinical Setting

#### Radiation Recall Pneumonia

**Figure 2:**
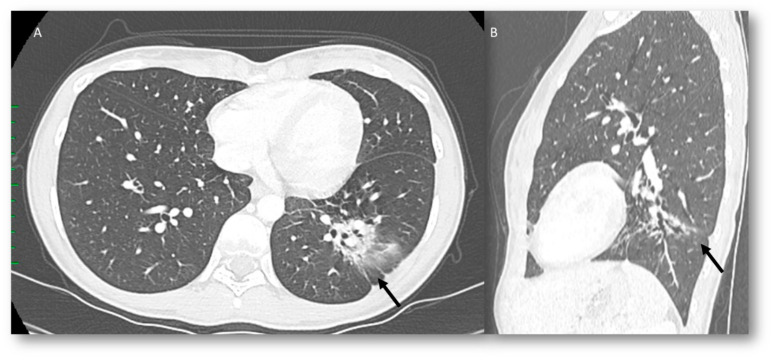
CT assessment ((**A**): axial plane) of RRP (arrow) in radio-treated melanoma metastasis during ICI therapy; resolved ((**B**): CT in sagittal plane) after steroid therapy (arrow).

Radiation recall pneumonia (RRP) and CIP show similar findings on CT scans; however, the distinctive element is the location. In fact, RRP CT findings include consolidative (Figure 2) or ground-glass opacities limited to a prior radiation field, and the main common radiological pattern is a cryptogenic organizing pneumonia (COP) [109]. It should be supposed in any patient subjected to radiation therapy with new airspace changes sharply delineated from the adjacent lung in the radiation field appearance. The main differential diagnosis is infection, which occurs outside of the prior radiation field [110].

**Figure 3:**
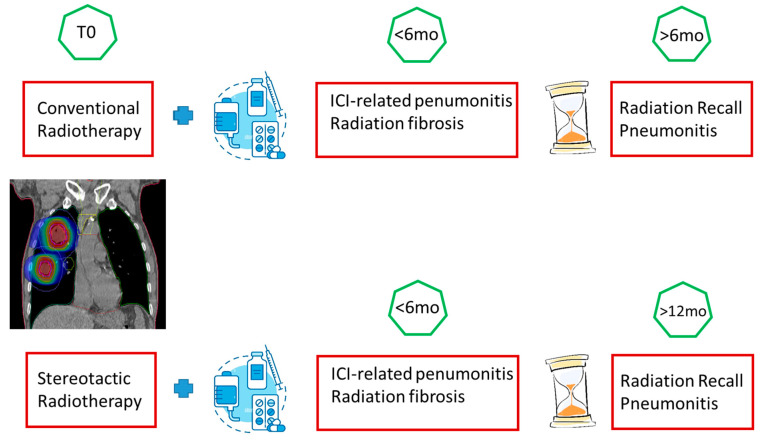
Graphical representation of the different pneumonia related to RT, considering the onset timing.

Cousin et al., in their retrospective study, evaluated the incidence, risk factors and CT characteristics of RRP in a cohort of 348 patients treated with ICI, of whom 80 had received thoracic radiotherapy previously. The purpose of the study was to identify diagnostic patterns that allowed the differential diagnosis between radiation pneumonia, immunotherapy pneumonia and radiation recall. They identified a time limit: RPP was defined as pneumonia that occurred 6 months after the previous conventional radiotherapy and a year after the previous stereotactic radiotherapy (SBRT). The time factor is crucial (Figure 3) given the similar radiological features between RP and RRP (homogeneous or patchy areas of ground-glass opacity or consolidation, progressing towards fibrosis) [111].

Lu et al. [112] also identify the time factor as a fundamental element for differential diagnosis. In their cohort, the overall rates of different types of pneumonia were, respectively, 8.2% (n = 16) CIP, 46.9% (n = 92) RP and 7.1% (n = 14) RRP. In this study, the major differences between the various post-treatment pneumonias concern their timing of onset and CT features. Indeed, RP usually happened less than 6 months after RT within or at the edge of the radiation field, but CIP had a wider range of CT manifestations and a longer time window. RRP was defined as inflammatory reactions in previously irradiated fields on chest CT after ICI administration and more than 6 months after TRT. As far as CT characteristics are concerned, the most characteristic results concern CIP with the finding of diffuse patches (47.4%), consolidation (26.3%) and ground-glass opacity (26.3%), unlike RP and RRP pneumonias which have similar chest CT with consolidation-type lesions and streaks within the irradiation field [112].

In both studies and in clinical practice, the severity of pneumonia is graded according to the National Cancer Institute Common Terminology Criteria for Adverse Events Version (CTCAE) 5.0.

The latest version of CTCAE was published in 2017, and thanks to these criteria, it is possible to standardize and classify the severity of adverse reactions to oncology therapy and assess the interruption or non-interruption of treatment [36].

### 3.2. Immune-Related Pneumonia

**Figure 4:**
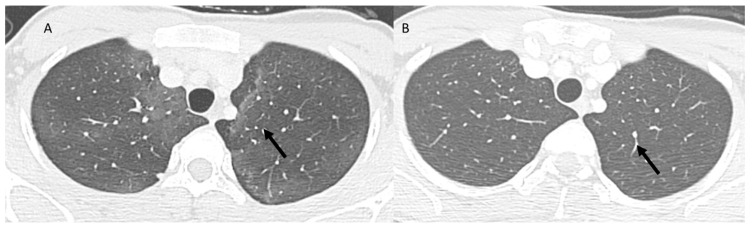
CT assessment (**A**) of CIP: OP pattern with peribronchovasal and subpleural distribution bilaterally of ground-glass opacities (arrow). Resolution of pneumonitis (**B**) after steroids (arrow) treatment.

Immune-related pneumonia (CIP) is an adverse event that occurs in 0 to 10% of cases after treatment with anti-PD-1/PD-L1 mAbs as focal or diffuse inflammation of the lung parenchyma and has very variable onset times from a few days to more than a year after the start of treatment [113,114]. The most frequently pattern is an organized pneumonia (OP) with peribronchovasal and subpleural distribution bilaterally, characterized by consolidated opacities, ground-glass areas (Figure 4) or a combination of both and circumferential consolidative opacity surrounding an interior area of ground-glass attenuation (reversed halo sign), with a prevalent localization to the mid-lower lobes [115]. Pulmonary nodules may also be present, generally with a diameter of less than 10 mm; however, in some cases, the nodules may be larger and with spiculated edges, imitating and entering into differential diagnosis with malignant nodules [115]. The second frequency pattern is non-specific interstitial pneumonia (NSIP), which occurs with ground-glass and reticular opacities with lower lobe predominance, while consolidative opacities are uncommon [116]. Airspace disease is synchronous and relatively symmetrical. The subpleural sparing of the posterior and lower lobes and the absence in most cases of consolidative opacities are characteristics that allow us to distinguish NSIP from the OP pattern.

**Figure 5:**
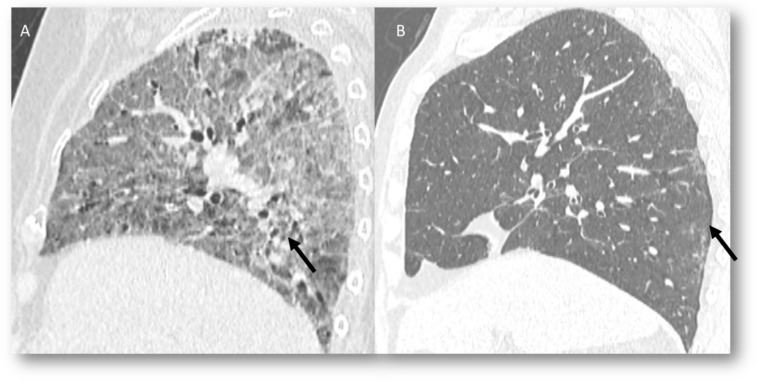
CT assessment ((**A**): sagittal plane) of CIP: acute interstitial pneumonia (AIP)–acute respiratory distress syndrome (ARDS) pattern (arrow). After steroids treatment ((**B**): sagittal plane); subpleural distribution bilaterally of ground-glass opacities (arrow).

Less frequent are the hypersensitivity pneumonitis (HP) pattern and acute interstitial pneumonia (AIP)–acute respiratory distress syndrome (ARDS) pattern (Figure 5) [117]. The HP model is associated with lower-grade symptoms and is characterized by diffuse or predominant ground-glass centrolobular nodules in the upper lobe, which may be related to air entrapment. This pattern should be distinguished from HP exposure, respiratory and follicular bronchiolitis and atypical infection. In addition, here, for differential diagnosis is important the anamnesis and occupational exposure in the first case, the smoking habit, connective tissue disease or autoimmune diseases in the second and laboratory investigations and response to therapy in the last [117].

Acute interstitial pneumonia (AIP)–acute respiratory distress syndrome (ARDS) is associated with a more severe clinical course but is not usually related to ICI therapy [89].

### 3.3. Radiation Pneumonia

**Figure 6:**
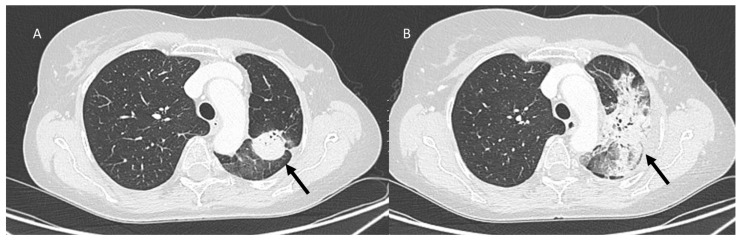
CT assessment of radiation pneumonia RP in RT-treated lung cancer. In (**A**), ground glass opacities (arrow) at about 8 weeks following completion of radiotherapy. In (**B**), consolidative pattern (arrow) at 12 weeks.

Radiation pneumonia (RP) typically occurs between 4 and 12 weeks following completion of radiotherapy; the most common results at chest CT are ground-glass opacities associated or not with areas of airspace consolidation, often with a halo sign or reversed halo sign (Figure 6). RP can occur as early density alterations (in 3–4 months), with a picture of pneumonitis or late when evolved into a fibrosis pattern (after 9 months) [118]. There is a current interest in imaging biomarker use to predict the RP risk. Several researches assessed pre-treatment [18F]-2-fluoro-2-deoxyglucose positron emission tomography (FDG PET) imaging towards this aim. The justification is linked to the idea that pretreatment inflammation would make pulmonary parenchymal more susceptible to treatment and hence RP [119,120]. Anyway, the open question is correlated to the diagnosis of RP in a setting of multimodality treatment, in particular when administrating immunotherapy. In fact, differentiating between RP and CIP has implications for clinical management; although the typical pattern of these entities is reported [121,122], these findings are only suggestive as pneumonitis can have a wide spectrum of appearance. In this scenario, artificial intelligence (AI) could allow proper patient management [123].

### 3.4. COVID-19 Pneumonitis and COVID-19 Vaccine Radiation Recall

Globally, on 23 December 2022, there were 651,918,402 confirmed cases of COVID-19, including 6,656,601 deaths. As of 22 December 2022, a total of 13,073,712,554 vaccine doses had been administered. At the end of 2022, according to the Italian Medicines Agency, COVID-19-related mortality on a global estimate stood at 0.045% compared to 1–2% when it debuted in our country [124].

**Figure 7:**
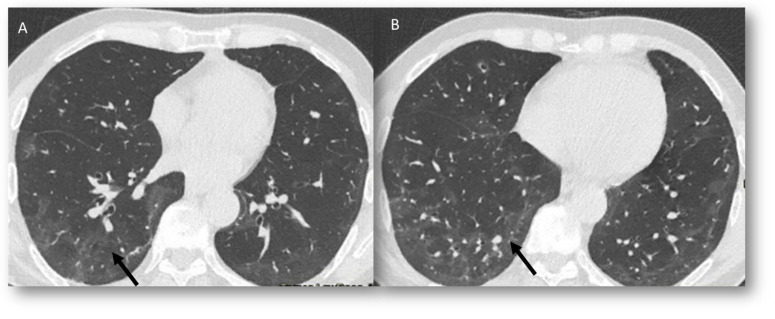
CT assessment of COVID-19 pneumonia (**A**,**B**): multifocal and bilateral distribution of ground-glass opacities (arrows).

On chest CT, the typical findings of COVID-19 pneumonia are the multifocal and bilateral distribution of ground-glass opacities (Figure 7) and consolidations and the thickening of the peripherally interlobular septa [125,126,127,128,129]. These findings go into differential diagnosis with the other patterns of pneumonia mentioned above for which they should be integrated with an RT-PCR test (that represents the gold standard for patients with ongoing COVID-19 pneumonia) or with an accurate history to exclude a previous infection [130,131,132,133,134,135,136,137,138,139,140,141,142,143,144,145,146,147].

**Figure 8:**
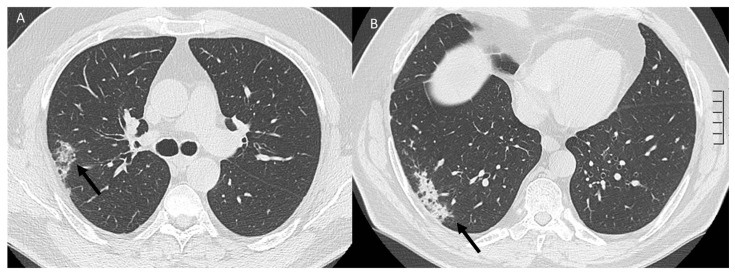
CT evaluation (**A**,**B**) of RRP induced by COVID-19 vaccine in RT-treated patients with lung metastases: consolidative pattern (arrows).

With regard to adverse reactions after vaccination for COVID-19, in the literature, there are several cases of RRP that see vaccination as a trigger (Figure 8). The latter, just like the COVID-19 infection, could determine an uncontrolled state of inflammation that favors the development of radiation recall [148,149]. On chest CT, scan areas of ground glass and consolidations are appreciated, but this time the localization in the same previously irradiated area allows us to make a differential diagnosis regarding the other infections. In this scenario and, considering the possibility that the patient is infected but without specific symptoms, proper management is crucial in order to avoid treatment discontinuation.

### 3.5. Progression Disease: Pulmonary Lymphangitis Carcinomatosa

**Figure 9:**
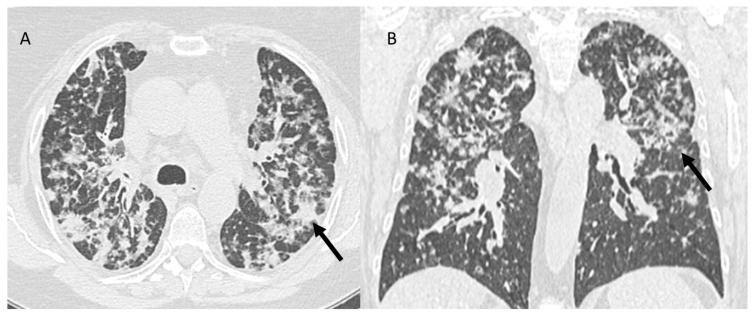
CT assessment ((**A**): axial plane; (**B**): coronal plane of PLC in pancreatic cancer patient: irregularly interlobular septal thickening or nodular thickening (arrows).

With regard to the involvement of pulmonary parenchymal, in an oncological setting, an accurate diagnosis of disease progression is crucial. An atypical lung involvement is the pulmonary lymphangitis carcinomatosa (PLC), which is a metastatic lung disease of malignant tumors that spread through pulmonary lymphatic vessels [150]. Although this condition may be considered rare, in patients with advanced disease status, it could be present. The primary cancers that are most frequently correlated with PLC are breast (17.3%), lung (10.8%) and gastroenteric (10.8%) cancers (Figure 9) [151].

Dyspnea is the main symptom, but often and especially in the early stages, it starts asymptomatically.

On CT images of the chest, the diffusion is variable; it can be unilateral or bilateral, localized or diffuse and is characterized by nodular or diffuse intrapulmonary infiltrates, irregular thickening of the interlobular septum, smooth thickening (initial stage) or nodular (late development), hilar and mediastinal lymphadenopathy and the opacity of ground glass. These features are also present in other interstitial lung diseases, thus having a low diagnostic specificity, but despite this, CT, and in particular high-resolution CT (HRCT), is the suggested technique for the study of patients with suspected PLC [151,152,153,154,155,156,157,158,159,160,161].

### 3.6. Machine Learning

Some promising tools in the diagnosis and follow-up of cancer patients who develop adverse reactions to treatments are artificial intelligence (AI) and radiomics [162,163,164,165,166,167,168,169,170,171,172,173,174,175,176,177,178,179,180,181,182,183,184,185]. Computers are able to accumulate and evaluate higher volumes of data compared to the human brain, so AI can resolve unsolved complexities in cancer patient management [162,163,164,165,166,167,168,169,170,171,172,173,174,175,176,177,178,179,180,181,182,183,184,185]. Machine learning (ML) is a sub-area of AI which uses mathematical algorithms and can learn specific tasks [162,163,164,165,166,167,168,169,170,171,172,173,174,175,176,177,178,179,180,181,182,183,184,185]. These models are supervised and unsupervised, depending on the knowledge of the desired outcome of interest [162,163,164,165,166,167,168,169,170,171,172,173,174,175,176,177,178,179,180,181,182,183,184,185]. The ML models analyze the input producing the necessary adjustments to obtain the desired output, or the models assess uncurated data and classify thanks to defined features within the dataset that can be grouped and analyzed further to reach a specific outcome, respectively, [162,163,164,165,166,167,168,169,170,171,172,173,174,175,176,177,178,179,180,181,182,183,184,185]. These models have made possible the development of a new approach named radiomics [162,163,164,165,166,167,168,169,170,171,172,173,174,175,176,177,178,179,180,181,182,183,184,185].

In addition, deep learning (DL), a form of AI, is becoming a promising support for medical imaging due to its capability of feature extraction and analysis [162,163,164,165,166,167,168,169,170,171,172,173,174,175]. It has been successfully employed in chest CT imaging to distinguish COVID-19 pneumonia from other infections and offer a qualitative and quantitative disease evaluation. With regard to adverse events, Giordano et al. [186], in their retrospective study, investigated if a deep learning algorithm was capable of discriminating COVID-19 from radiation therapy-related pneumonitis. They saw that the deep learning algorithm showed good sensitivity in recognizing cases of COVID-19 pneumonia and treated-related pneumonia (RP) but with a very low specificity (2%); in fact, almost all patients with RP were classified as suspected patients with COVID-19 pneumonia. However, specificity improved; instead of a binary analysis, a risk analysis was carried out with a 30% cut-off rate, whereas CT images of the chest were associated with a low risk of COVID-19 disease; above this threshold, patients were at high risk. In addition, this study supports the idea that deep learning algorithms based solely on CT images do not allow us to make differential diagnosis with high specificity between COVID-19 pneumonia and other interstitial lung diseases with very similar CT patterns [186]. Therefore, adding clinical and laboratory data to the assessment of the algorithm should improve the diagnostic performance. The algorithm demonstrated a good performance in CT finding segmentation, including ground-glass opacities, in order to help radiologist practice. However, as reported by the authors, with these unclear CT patterns and especially in oncological settings in which more treatments are associated and each one could to be responsible for pneumonitis, a multidisciplinary approach based on clinical history is mandatory.

## 4. Discussion

Knowing the CT features correlated to RT treatment is crucial to avoid confusing results and discontinuing treatments that are essential to the patient’s survival; although several features are typical, for example, PLC, COVID-19 and ICI-related pneumonitis show a diffuse parenchymal involvement, while RP and RRP are usually confined to the target volume. However, during COVID-19 infection, at the first phase, the parenchymal involvement could be restricted to a single lobe. In addition, PLC pneumonia shows an irregularly interlobular septal thickening or nodular thickening, while septal thickening is common in COVID-19 pneumonitis, RP and RRP. These features, during CT assessments, are the main findings for a proper diagnosis (Table 1). However, differentiating the dissimilar lung pathologies, based only on imaging assessment may be difficult, mainly during the pathologies’ early or late phases. Therefore, despite several characteristics, they could orient towards one or the other pneumonia; the differential diagnosis of the various lung diseases still represents a great challenge for the radiologist and the multidisciplinary team. In fact, when the cause of the lung involvement is ambiguous, as CT findings are unspecific, multidisciplinary management is necessary to establish the proper treatment. Although oncological patient management should contemplate the possibility of disease progression or adverse effects, in pandemic conditions, an undetected infection and the possibility that COVID-19 vaccine may be a trigger for RRP should also be considered. Therefore, the clinical data evaluation, based on the medical and pharmacological history, analysis of radiological imaging and assessment of the response or not to proceed with therapy, have a fundamental role in the classification of the patient’s lung disease.

In the near future, further help with differential diagnosis will come from the use of AI software and the analysis of radiomics features; although the reported literature data showed a great AI potential in pneumonitis diagnosis. However, most of the published papers are experimental, and the analysed data sets suffer from selection bias as symptomatic patients are included. Moreover, most studies employed imbalanced data sets. Therefore, the reported performance of various AI algorithms employed may have been affected by polarization of the context. More effort is required to handle imbalanced data sets prior to the application of AI. In addition, the assessed CT features were from patients with severe disease. A larger database, which includes patients at different stages of disease, is required to optimize the diagnostic system.

## 5. Conclusions

The management of oncological patients, subjected to combined therapies, is challenging, especially in treatment-adverse event setting. Knowledge of pneumonia related to cancer therapy is critical, as often the CT findings are similar. In this scenario, the AI software and the analysis of radiomics features could help multidisciplinary team diagnosis.

## Figures and Tables

**Table 1:** Lung involvement and CT pattern for pneumonia type.

Type of Pneumonia	Lung Involvement	CT-Patter	Mechanisms of Lung Radiation Damage
RRP	Target area	Ground-glass opacities and consolidative opacities.	Unknown (A non-immune fixed drug reaction-like condition, dysregulated release of reactive oxygen species, abnormalities of tissue vasculature and impaired DNA repair).
RP	Target area	Ground-glass opacities and consolidative opacities.	Direct damage to the DNA and indirect damage through the production of reactive oxygen species (ROS), causing changes in vascularity and capillary permeability, activation of the inflammatory response and alteration of immunological response
ICI-related pneumonitis	Diffuse (related to the phase of disease)	Ground-glass and reticular opacities; consolidative opacities; interlobular septal thickening; “crazy-paving” pattern	Autoimmune
COVID-19 pneumonia	Diffuse (related to the phase of disease)	Ground-glass opacities; crazy-paving pattern; consolidative opacities; interlobular septal thickening (according to the phase of disease)	Unknown, supposed cytokine storms
Pulmonary lymphangitis carcinomatosa	Diffuse (related to the phase of disease)	Irregularly interlobular septal thickening; smooth (early stage) or nodular thickening (late development); ground-glass opacities; pleural effusions.	Tumor spread through lymphatic vessels

## Data Availability

All data are reported in the manuscript.
